# Clinical practice enhanced by interdisciplinary theoretical perspectives

**DOI:** 10.1080/02813432.2024.2368852

**Published:** 2024-06-19

**Authors:** Kirsti Malterud, Susanne Reventlow, Ann Dorrit Guassora

**Affiliations:** a The Research Unit for General Practice and Section of General Practice, Department of Public Health, University of Copenhagen, Copenhagen, Denmark; b Department of Global Public Health and Primary Care, University of Bergen, Bergen, Norway

**Keywords:** Clinical medicine, general practice, interdisciplinary theories, experience-based knowledge, evidence-based practice

## Abstract

**Background:**

Experience-based knowing in general practice includes advanced interpretation of subjective, complex and particular phenomena in a social context. Enabling different metapositions for reflexivity may provide the accountability needed for such knowing to be recognized as evidence-based practice.

**Objective:**

To demonstrate and discuss the potential of substantive theories to enhance interpretation of complex challenges in clinical knowing in general practice.

**Methods:**

We present a fictional case to demonstrate how interdisciplinary substantive theories, with a relevant and specific match to concrete questions, can situate the clinical interaction at an accountable platform. A female patient with Parkinson’s disease consults her GP complaining that the disease is restraining her life and threatening her future. The GP has some new ideas from Bandura’s theory of self-efficacy and introduces the patient to strategies for further action.

**Findings:**

The case presents an example of how a relevant substantive theory may offer the GP: 1) a sharper focus for achievement: recognising the issues of fear and identity in chronic, progressive illness, 2) a subsequent position for individualized understanding of adequate strategies: encouraging physical and social activity in a well-known context, and 3) an invitation to consider further possibilities: finding ways to alleviate the burden of fear and progressive decline; engaging in joyful living.

**Implications:**

General practice knowledge embraces a diversity of sources with different evidence power. The transparency mediated to clinical practice when supported by relevant substantive theories may contribute to recognition of experience-based knowing as evidence-based practice.

## KEY POINTS

- Experience-based knowing is an important capacity for interpretation of complex phenomena in general practice, but support for elaboration and analysis of such strategies is limited
- Interdisciplinary substantive theories can enable reflexivity and enhance clinical practice
- Substantive theories may offer sharper focus, individualised understanding and recognition of additional possibilities
- A connection to specific theoretical ideas may augment the transparency of experience-based knowing, thus advancing attitudes for evidence-based practice

## Background

The clinical encounter between doctor and patient in the context of family, society and culture, constitutes general practice [1]. Within this medical domain, biomedical and epidemiological models are necessary but often insufficient, when clinical reasoning incorporates the complex challenges of diagnosis, treatment and interaction with the individual patient [2]. Developing clinical knowing, the general practitioner (GP) applies advanced interpretation of subjective phenomena, such as perceptions of bodily signs and symptoms, human interaction and values [3–5]. This kind of expertise tends to be experience-based and tacit [6], deserving particular attention for the development, maintenance and contestation of professional standards beyond gut feelings [7].

Schön conceptualized experience-based knowing as an unarticulated conversation between the expert and the situation and called it *reflection-in-action* [8]. To some extent, reflection-in-action may be transformed to *reflection-on-action* by self-observation, communication and analysis, using qualitative methods (see for example [9]). In this article, we take Schön’s perspectives both as our preconception and our analytic approach, exploring interpretation in clinical knowing as it is instantly developed and applied by the GP in the consultation [6]. We shall introduce additional concepts and tools suggesting potentials for enhancing the experience-based aspects of clinical knowing.

## Reflexivity - indispensable for clinical practice

All knowing is determined by interpretation. Haraway proposed *situated knowing* as a term implying that knowledge is always partial, located in a specific position dependent on the view and interests of the knower [10]. Situated knowing is an appropriate concept for clinical knowing in general practice, referring to the knowers’ responsibility to recognize their own interpretative position and to acknowledge the limitations of any knowing.

*Reflexivity* addresses the accountable role of the knower. The concept was first coined in the early 1990s by qualitative researchers and social constructionists. Reflexivity was presented as an attitude recognizing ‘the research process itself as … socially constructing a world of worlds, with researchers included in, rather than outside the body of their own research’ [11] (pp. 2–3). Reflexivity implies ‘bending back’ toward an awareness of the role of the researcher and the context of the research as issues to be shared and disputed as social processes. At the same time, clinicians inspired by anthropology promoted a similar notion of reflexivity, as ‘a self-conscious account regarding the condition of knowledge production as it is being produced’ [12]. Baarts et al. presented a case illustrating how the GP reflexively seeks to understand the patient’s values, norms and perceptions of the situation, while simultaneously distancing herself to look objectively at signs. Reflexivity wakes up the responsibility of the knower, insisting on an awareness of the situated nature of interpretation and knowing. A reflexive attitude may hence offer paths for unravelling experience-based clinical knowing and support professional accountability and quality.

*Metapositions,* where people step aside and have a look at the reality in which they participate, invite professionals to reflect upon their clinical work [13], hence metapositions enhance reflexivity in interpretation [14]. Theoretical perspectives can serve as metapositions, contributing not only to inspiration, but also to transparency. Below, we shall introduce substantive theories, suggesting how this specific type of theory can enhance reflexivity in clinical knowing.

## Theories supporting interpretation, reflexivity and knowing

A *scientific theory* is a consistent, soundly based set of assumptions about a specific aspect of the world, predicting or explaining a phenomenon [15]. Medical doctors are familiar with theories from the natural sciences and epidemiology, indicating predictability, verification and falsification. In daily practice, such theories are therefore often taken for granted, as implicit facts in no need of argument. In a slightly different mode, we will draw attention to how theories from the humanities and social sciences can offer concepts, models and chains of arguments with the potential to enhance a GP’s interpretation and handling of complex clinical challenges on the go.

*Philosophies* (such as phenomenology or social constructionism), and *formal theories* (such as Freud’s psychoanalysis or Maslow’s hierarchy of human needs) can be helpful for medical practice. Here, we want to introduce *substantive theories* - a level of scientific theory that provides more practicable metapositions for clinical practice by relating to concrete issues, experiences or activities. Such theories have been applied in everyday contexts, for example, in social and educational sciences, linguistics and economic geography. We have elaborated the concept ‘substantive theories’ (in Danish: temateorier) further while developing qualitative research methods in general practice, as theories with a relevant and specific match to concrete questions in our research projects [16]. Our clinical background as GPs gradually led us to recognize the potential value of substantive theories beyond research, as metapositions for knowing in clinical practice [14], mediating practical ideas as well as transparency.

## Objective

The aim of this article is to demonstrate and discuss the potential of substantive theories to enhance interpretation of complex challenges in clinical knowing in general practice.

## Method

For this purpose, we present a fictional case to illustrate the capacity of a specific substantive theory in an everyday clinical situation in general practice. Drawing on our own clinical experiences, complemented by reflections after a group discussion with three GPs, we developed the case story. The patient is Elsie, aged 63, who was diagnosed with Parkinson’s disease two years ago, and her GP, Mark, aged 48. Elsie is a former hotel manager, and for the last year she has been receiving a disability pension. She has been on Mark’s patient list for ten years.

The case story will exemplify how a specific substantive theory supports a GP to interpret a unique situation and tailor individual strategies for a patient living with chronic, progressive illness. Representing a metaposition, the theory situates the clinical interaction at an accountable platform, without confining the approach into a deductive standard.

We will first stage the case descriptively as we imagine the GP perceived it. Then we present our reflections and analysis upon the case. Finally, we sum up the main findings of our analysis.

### The case history

Elsie is sitting in a tall chair in the waiting room. Mark, the GP, greets her and waits for her to get up. Elsie used to be elegantly dressed, but not today. She walks slowly, and her left leg trembles a lot. The reason for the visit was listed in the GP’s schedule as ‘Mood’. Walking to the consultation room the GP notices that Elsie has a couple of notes in her left hand. One of them looks like a medication list.

In the consultation room Elsie says that she feels stuck and worries a lot. The first year after her diagnosis with Parkinson’s disease, she was relieved that medicine helped; but now she is experiencing deterioration. Mark looks up the notes from Elsie’s last check-up with the neurologist and the speech therapist, reporting Elsie’s condition as more or less stable with a slow loss of function, as expected. There have been no sudden changes. Still, Elsie is clearly more burdened now than she was a few months ago. The GP asks about Elsie’s everyday life, and Elsie says that she has enjoyed the company of an old friend from school, who also has Parkinson’s disease. They have talked about a lot of things that Elsie does not want to burden her husband or her children with. Now, her friend has become too ill to take the bus to visit her, and Elsie misses those visits a lot. Elsie used to attend the Parkinson’s patients’ association, but the activities they offer no longer appeal to her.

Elsie’s energy levels seem to be more or less as usual, and she eats and sleeps as before. Knowing Elsie over years, Mark recalls his previous knowledge from earlier encounters. Mark considers whether Elsie is depressed but rejects this diagnostic possibility after further dialogue.

An idea comes to his mind, linked to the notion of self-efficacy, which he briefly heard about some months ago. Mark does not remember the details, but he picked up the main message that self-efficacy is boosted by experiences of mastering challenges and can enhance confidence by being able to do something more. He takes into consideration how it might be possible to involve Elsie in such ideas. He recalls that Elsie enjoyed the dancing evenings from her time as a hotel manager and says: ‘You used to enjoy dancing. Do you see any possibility that you can dance again?’. Elsie replies reluctantly that it has been a long time since she last went dancing. However, she admits, the patients’ association organize dance evenings, but she has neither courage nor desire to attend. Talking more about this, Elsie gradually changes her mind and says she will try it out and see whether this kind of dance will suit her.

Coming back a month later, Elsie says she was surprised to learn that by dancing, her body works a lot better than she thought it would. Her mood is much improved by that experience, and it has also helped her to dare travelling all the way home to visit her school friend. They have met several times and enjoy each other’s company again.

### Reflections - metapositions for clinical practice offered by substantive theories

Having described the GP’s perspective, we now shift toward a reflexive mode where we strive for reflection-on-action, exploring the potential relevance and impact of substantive theory [8]. We apply this position and attitude for a second order analysis [17] aiming to identify and articulate relevant metapositions for interpretation offered to the GP by a specific substantive theory (Bandura’s theory about self-efficacy [18]).

But first a brief summary of this theory: *Self-efficacy* is a person’s belief that they can give rise to a change. Bandura, a psychologist, demonstrated how strategies to strengthen self-efficacy in people with phobic behaviour had a strong impact on their capacity to accomplish the required behavioural changes. His theory implies that a person’s self-efficacy can be enhanced by successful experiences, support from other people or observation of other people’s experiences. Our case story exemplifies a substantive theory about self-efficacy as inspiration for a GP to tailor individual strategies for a patient with chronic, progressive illness.

Drawing on experiences from the long-term relationship, the GP, Mark, knows the patient, Elsie, as a resourceful and well-organized person who used to face her gradually increasing symptom burden quite well. Walking together from the waiting room to the office, he senses her impaired mobility and resigned look. Elsie presents symptoms suggesting a depression and talks about the progress of her Parkinson’s disease as a threat to her future. Mark knows that Elsie has no previous history of depression, so he rather tries to sort out the daily impact of her neurological symptoms. Elsie’s actual burden then seems to be a feeling that her everyday life is jeopardized. Mark assumes, therefore, that antidepressant medication and psychotherapy will not make a positive change.

Taking some elements from Bandura’s theory about self-efficacy as a point of departure, Mark then arrives at specific experiences and paths to pursue that provide him with ideas for concrete strategies to support Elsie’s self-efficacy. Deliberate changes of behaviour, customized from Elsie’s personal experiences and capacities, might have the potential to improve her perception of herself as a person and prevent her fear for the future, instead of letting her fear drag self-efficacy downward.

Mark knows the meaning of self-efficacy, although he is not familiar with the details of the empirical evidence. In the consultation, he interprets Elsie’s actual trouble as low self-efficacy coming out of reasonable fear. He follows no checklists or guidelines. His experiences and relationship with Elsie and his knowledge of her history make him recognize her as a patient with a rich resource bank of challenging as well as positive experiences. Following the encouragement of the self-efficacy metaposition, Mark navigates toward domains of Elsie’s life where she has previously experienced mastery. He knows that his support can be valuable, since Elsie trusts him from previous encounters over ten years. Mark suggests that she takes up dancing again, but now with other patients with Parkinson’s disease. He expects her self-efficacy to increase by seeing others in similar health circumstances master challenges, which she may overcome in this particular context of dancing.

### Analysis: the impact of substantive theories as metapositions in clinical practice

In summary, we find that Mark applies the theory of self-efficacy on a level that offers a few, manageable strategies supporting his interaction with Elsie. The theory provides him with a sharper *focus* for achievement (recognising the issues of fear and identity in chronic, progressive illness); allows him a subsequent position for individualized *understanding* of adequate strategies (encouraging physical and social activity in a well-known context); and invites additional *possibilities* (finding a way to alleviate the burden of fear and progressive decline; engaging in joyful living).

Our aim was, however, neither limited to Bandura’s theory nor to Parkinson’s disease. Below, we shall use these findings to discuss strengths, limitations and transferability of metapositions offered by this and other substantive theories in the general practice context.

## Discussion

Presenting the case story, we established a clinical context where a patient with a chronic, progressive disease told her GP about her distress related to the gradually increasing symptom load. We introduced a substantive theory (Bandura’s theory of self-efficacy) that came to the GP’s mind, offering him new ideas that added to his previous experience and the long-term relationship he had with this patient. Reflecting on the course of the case story, we identified sharper focus, individualized understanding and new possibilities as outcomes emerging from the GP’s inductive application of this substantive theory. Below, we discuss the impact of these interpretations and some limitations and strengths of our approach.

### What is known from before – what does this study add?

We have presented a specific strategy where adequate substantive theories are introduced and applied in everyday practice. The strategy is intended to add to existing aspects of EBM, rather than suggesting an entirely new approach. Our case illustrates how relevant theories responsibly can inform, expand and strengthen experience-based knowing. We are still working on elaboration and development of educational tools to implement the strategies presented above.

GPs are available for any health problem in a person at any age over time in a specific social context. The variety of inquiries and problems is diverse, and often not presented as well-defined disease units. Patients present symptoms in the very early stages of potential disease development, and multimorbidity blurs the diagnostic gaze as well as the therapeutic options. The GP’s clinical experience combined with the continuity of the doctor-patient relationship are important sources for interpretation of complex clinical challenges [1,19]. We suggest theoretical concepts and models to complement the biomedical and epidemiological knowledge base for clinical practice, but we are not the first.

Almost 50 years ago, Engel portrayed the limitations of the biomedical model and proposed a new *biopsychosocial medical model* based on a general systems theory perspective [20]. Engel’s model applied a contextual understanding, including the subjective elements of health and disease. This model has mostly functioned as a formal, overarching theory, reminding clinicians that biology exists as part of a larger system of complex knowledge, without providing more specific approaches to the diversity of clinical problems. Introducing substantive theories as a diverse reservoir of ideas complementing the GP’s experience, we offer tools to understand how individualized strategies may be tailored within a biopsychosocial understanding.

The *patient-centred clinical method*, developed by McWhinney and colleagues from the early 1980s [21], takes values from the biopsychosocial medical model as a platform for clinical work in general practice. These perspectives have had a significant impact on the foundation and teaching of this medical specialty, primarily highlighting consultation skills and emphasizing the situation and context of the individual patient. Narrative medicine [22] has also provided overarching standards for clinical interaction resonating well with patient-centredness. Substantive theories may further support the GP in focusing the problem and seeing possibilities in patients’ particular situations. In this way, more options for relevant interaction and action may be developed.

In 2011 the Institute of Medicine (US) published a set of standards for developing rigorous, trustworthy *clinical practice guidelines* (CPG) as a systematic aid to making complex medical decisions [23] (pp ix-x). The aim was to reduce inappropriate practice variation, enhance translation of research into practice, and improve healthcare quality and safety. Several guidelines are used in general practice, but GPs often find that the principles of standardization underlying this knowledge base, developed at a group level, limit implementation with the particular patient. A wide span of practicable possibilities, strategies and solutions may appear when interdisciplinary substantive theories complement evidence-based strategies.

### Experience-based knowing in evidence-based medicine?

**Figure 1.**
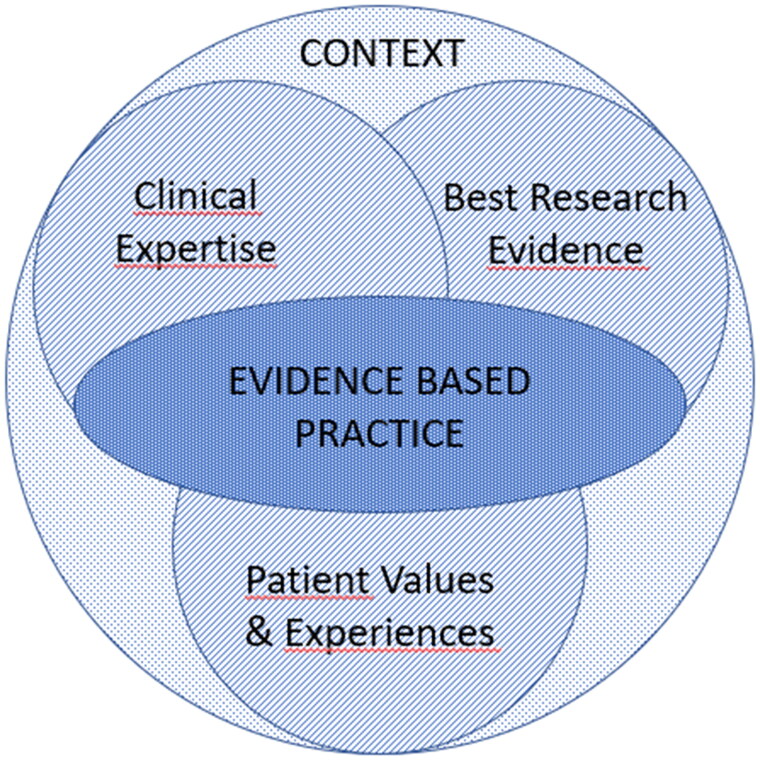
Evidence-based Practice (after [24,25]).

As medical specialists, GPs are accountable to values of *evidence-based medicine* (EBM), with integration of knowledge from best available external research evidence, individual clinical expertise, and patient values and experiences (Figure 1) [24,25].

Essential aspects of general practice are increasingly documented by research evidence developed with both quantitative and qualitative methods. Patient values and experiences are incorporated by patient participation and shared decision-making. But what counts as evidence in the domain of clinical expertise and experience-based clinical knowledge? How can this mode of knowing come into view as something beyond random whims or unfounded habits [6]?

Sackett, one of the founding fathers of EBM, wrote:

Individual clinical expertise incorporates the proficiency and judgment that individual clinicians acquire through clinical experience and clinical practice. Such expertise is reflected as more effective and efficient diagnosis and in the more thoughtful identification and compassionate use of individual patients’ predicaments, rights, and preferences in making clinical decisions about their care. [25]

Taking the broad standards of evidence-based practice seriously, we suggest reflexivity as a gold standard for attributing the ‘evidence label’ to experience-based knowing in clinical practice [11]. A substantial proportion of experience-based knowing will always take place as unarticulated reflection-in-action. Still, practitioners can be encouraged toward reflection-on-action through sharing and reflecting upon useful experiences with colleagues [8]. Opening a window to the foundation and processes of knowing is a significant contribution to transparency, as making experiences available for talk and discussion is an act of reflexivity. But reflexivity also works by enriching and connecting existing habits and skills with relevant substantive theories, serving as metapositions for further reflection and commitment.

Clinical expertise includes a diversity of accumulated experience-based knowledge. A theory is useful if it gives GPs a new idea or direction that connects with, and adds to, their experience, supporting the GP with focus, understanding, and new possibilities which lead to strategies and action. Familiarity with and knowledge of concrete substantive theories can thus contribute to the content of clinical work in general practice in encounters with patients.

The GP in our case could certainly have proceeded adequately without Bandura’s theory coming to his mind. We wanted, however, to demonstrate how the perspectives from a substantive theory added to the doctor’s own experiences by introducing new perspectives for interpretation resulting in possible new strategies. Our case also exemplifies how this process may take place in an inductive mode, as opposed to a pre-planned, deductive checklist approach. A detailed competence of self-efficacy or the empirical basis was not necessary for GP Mark to benefit from Bandura’s ideas. In this way, substantive theories can contribute in a systematic way to expand and support the content of experience-based knowledge.

### Strengths and limitations of our approach

Developing the case, we took a lot of efforts considering how a commonly occurring clinical challenge could incorporate and illustrate aspects of transferability to contexts beyond the particular patient, doctor, diagnosis and theory. We chose to present an elderly patient with a chronic, progressive disease, for which a lot of biomedical knowledge, epidemiology and guidelines already exist. Encountering a young patient with a more fluctuating and unpredictable diagnosis, the doctor could as well have called for new ideas to interpret the situation and suggest some practicable strategies, possibly represented by some other theoretical perspectives and concepts. From a large toolbox of substantive theories, we could have illustrated a different case with other substantive theories, arbitrarily chosen and relevant for the actual problem, for example Lakoff & Johnson’s metaphor theory (whenever people interpret an image, experience, or event expressed by another word or concept, they are using metaphors) [26], Antonovsky’s salutogenesis (encouraging a shift of attention from illness to disease, from risks to resources) [27] or Star & Griesemer’s boundary objects (translations, negotiations and simplification of concepts to unify perspectives and obtain common understanding and collaboration) [28]. Considering several different substantial theories in relation to a specific problem should be encouraged, since they can make the problem appear in different ways and hence inspire different understandings. It is also a point that different clinical problems deserve reflection from different substantive theories, emphasizing that there are much more to substantial theories than Bandura.

Our case portrayed a GP with a rich reserve of experiences, both as a GP and from this long-term relationship with the actual patient. He still found that a substantive theory to give additional, specific value to his interpretation, offered some new directions and also a window of transparency. For a less experienced doctor, the theory would perhaps be even more influential as a building brick in his or her foundation wall. In another relation, with more tensions, new ideas might bring some creative surprises, allowing for diversions from how things usually passed on.

Finally, some words of caution: A substantive theory will not by itself solve any problem, and sometimes it does not fit the situation at all. Even with a comprehensive theoretical framework in place, there will always be a multitude of challenges for the GP.

## Implications for clinical practice

Illness and health are dynamic processes embedded in and shaped by the sociocultural context. General practice knowledge must therefore incorporate a diversity of knowledge and evidence sources. Demonstrating the relevance of theories from the humanities and the social sciences, we may contribute to expand the understanding of medical problems.

Theoretical perspectives are often ambiguously embedded in experience-based clinical knowing. Reflection-on-action can make theory at play more tangible and accountable. Adding interdisciplinary substantive theories as tools for knowing and reflexivity can further enhance clinical practice by enabling the GP to navigate the complexities of clinical problems. Advancing substantive theories, general practice knowing can be enriched with a broader understanding, ultimately empowering the GP to better serve the particular patient, while supporting transparency and evidence-based attitudes in the process of knowing.

## Ethical approval

For reasons of confidentiality, we constructed the case by assembling medical and demographic details from several instances, arguing that a consultation with similar elements of knowing is both familiar and frequently occurring in general practice. All procedures performed in studies involving human participants were in accordance with the ethical standards of the institutional and/or national research committee and with the 1964 Helsinki declaration and its later amendments or comparable ethical standards. Since the article does not contain studies with actual human participants, informed consent and approval from The Regional Committees for Research Ethics were not relevant.
